# Association Between the Normal-Density Psoas Muscle Index and Handgrip Strength or Gait Speed in Maintenance Hemodialysis Patients

**DOI:** 10.3389/fmed.2021.712497

**Published:** 2021-07-28

**Authors:** Byung Hoon Kwack, Jun Chul Kim, Jun Young Do, Seok Hui Kang

**Affiliations:** ^1^Department of Orthopedic Surgery, Soonchunhyang University Gumi Hospital, Gumi, South Korea; ^2^Division of Nephrology, Department of Internal Medicine, CHA Gumi Medical Center, CHA University, Gumi, South Korea; ^3^Division of Nephrology, Department of Internal Medicine, College of Medicine, Yeungnam University, Daegu, South Korea

**Keywords:** psoas muscle, hemodialysis, muscle strength, gait speed, fatty change

## Abstract

**Introduction:** This study aimed to evaluate the association between the psoas muscle (PM) index with or without fatty infiltration and various indices associated with muscle mass in hemodialysis (HD) patients.

**Methods:** We included stable HD patients (*n* = 83). The collected data included subjective global assessment (SGA) score, ASM/Ht^2^ (appendicular skeletal muscle mass divided by the squared height in meters), gait speed (GS; m/s), and handgrip strength (HGS; kg). The abdominal computed tomography (CT) image was obtained using a CT scanner. The PM and normal-density PM (NPM) indices (mm^2^/m^2^) were calculated using the whole PM area or the area with an attenuation range of 35–100 HU divided by the squared height in meters.

**Results:** Correlation coefficients for the SGA score, ASM/Ht^2^, HGS, and GS were greater for the NPM index than for the PM index. The linear regression analysis showed that, on multivariate analysis, the NPM index was significantly associated with the SGA score, ASM/Ht^2^, and GS. However, the PM index was significantly associated with the SGA score and ASM/Ht^2^ but not with HGS or GS. For calculating the low GS, the area under the receiver operating characteristic curve area was significantly greater for the NPM index than for the PM index (*P* = 0.012).

**Conclusion:** The present study suggested that the NPM index excluding fatty infiltration may be an early and useful indicator for detecting muscle strength and physical performance among HD patients.

## Introduction

Hemodialysis (HD) is one of most commonly used dialysis modalities in end-stage renal disease patients requiring renal replacement therapy. With advances in HD technologies and cares, the survival of HD patients has improved. However, the prevalence of long-term complications increased over time with treatment and as the dialysis population ages (1). Decreased muscle mass is one complication that can develop in long-term HD patients (2). This is associated with decreased quality of life and increased morbidity or mortality in HD patients (3). Therefore, accurate muscle mass measurement is important for predicting prognosis or physical performance in HD patients.

Computed tomography (CT) and dual X-ray absorptiometry (DXA) are commonly used for measuring muscle mass in HD patients (4, 5). Previous studies have shown that, among various muscle mass indices, the psoas muscle (PM) index measured on CT is associated with clinical outcomes in HD patients (6, 7). However, HD patients are prone to insulin resistance caused by various conditions such as diabetes mellitus (DM), chronic inflammation, or uremia (8). Insulin resistance can be associated with fatty changes in the muscle mass in HD patients and lead to overestimated muscle mass measurements. Both PM including or excluding fatty infiltration can predict the total muscle mass index and nutritional index. The two indices are positively correlated with total muscle mass. Both muscle mass and fatty infiltration can be increased by improving nutrition in dialysis patients. However, the predictability of muscle strength or physical performance differs between the two indices. Although the total amount of muscle mass was to be the same, muscle mass with fatty infiltration is associated with a greater decrease in muscle strength or physical performance than that without fatty infiltration. These reveal that muscle mass without fatty changes may be more closely associated with muscle strength or physical performance than total muscle mass *per se*. However, there are few studies of the association between muscle without fatty changes and indicators associated with muscle function in HD patients. This study aimed to evaluate the association between the PM index with or without fatty infiltration and various indices associated with muscle mass in HD patients.

## Materials and Methods

### Study Design and Participants

Our study was a cross-sectional study and it was performed between September 2012 and March 2015 in a medical center. We included HD patients aged ≥20 years and with duration of dialysis ≥6 months, ability to ambulate without an assistive device, ability to communicate with the interviewer, and no hospitalization within the 3 months before enrollment. This study was approved by the institutional review board of a local medical center (approval no. 12-07). Informed consent was obtained from all participants before enrollment: a total of 83 patients were enrolled. None of the participants took medications associated with physical activity such as opioids, antihistamines, or antidepressants.

### Baseline Variables

The collected baseline data included sex, age, presence of DM, dialysis vintage, hemoglobin (g/dL), C-reactive protein (CRP; mg/dL), blood urea nitrogen (mg/dL), creatinine (mg/dL), calcium (mg/dL), phosphorus (mg/dL), sodium (mEq/L), potassium (mEq/L), chloride (mEq/L), intact parathyroid hormone (i-PTH; pg/mL), total cholesterol (mg/dL), albumin (g/dL), and single pool measurement of solute removal during HD that focuses on urea (spKt/V_urea_). All laboratory tests were performed prior to the HD sessions and repeated thrice in the following 3 weeks. The mean of three values was recorded for each parameter. DM was defined as a patient-reported history and medical record of a DM diagnosis or use of antiglycemic medication. The spKt/V_urea_ values were calculated using the described as described previously (9).

### Assessment of the Nutritional Status, Muscle Mass, Strength, and Gait Speed

The subjective global assessment (SGA) score was calculated based on scores from seven items (weight loss, dietary intake, gastrointestinal symptoms, functional capacity, comorbidity, decreased fat, and decreased muscle mass) as described previously (10). Muscle mass was evaluated using whole-body DXA (GE Medical Systems Lunar, Madison, WI, USA). Appendicular skeletal muscle mass (ASM; kg) was calculated using the sum of lean mass of both extremities. Finally, ASM/Ht^2^ was calculated as ASM divided by the squared height in meters. Hand grip strength (HGS) was measured and each patient performed three trials with the dominant hand using a manual hydraulic dynamometer (Jamar® Hydraulic hand dynamometer; Sammons Preston, Chicago, IL, USA). Maximum values among the three trials were selected. Gait speed (GS; m/s) was evaluated using the timed 4-m walking test (11). A low GS group was defined based on their values <0.8 m/s, according to a previous guideline (11). We also defined the low SGA (<6 of total SGA score), low ASM/Ht^2^ (<7.0 kg/m^2^ for males and <5.4 kg/m^2^ for females), and low HGS (<26 kg for males and <18 kg for females) groups based on the same guidelines (11).

### PM Evaluation

The abdominal CT image was obtained using a 320-slice CT scanner (Aquilion ONE; Toshiba Medical Systems Corp., Tokyo, Japan). A single axial image was obtained at the lower border of the L3 vertebral level. The images were analyzed using an image analysis software (ImageJ 1.45S; National Institutes of Health, Bethesda, MD, USA). In our study, Hounsfield units (HU) of muscle were defined using a modified version described previously (12). Muscle attenuations at very low, low, or normal density were −29 to −1, 0–34, and 35–100 HU, respectively. A quantitative assessment of the whole PM was obtained and PM area (mm^2^) was defined as the area with an attenuation range of −29 to 100 HU among the sum of the right and left whole PM areas, which includes very low-, low-, and normal-density muscle. The PM index (mm^2^/m^2^) was calculated using the PM area divided by the squared height in meters. Normal-density PM (NPM) area was defined as the area with an attenuation range of 35–100 HU among the sum of the right and left whole PM areas including normal-density muscle alone. The NPM index (mm^2^/m^2^) was calculated using the NPM area divided by the squared height in meters. Three measurements were taken of both PMs and the average was used for the analysis.

### Statistical Analysis

The data were analyzed using IBM SPSS Statistics version 25 (SPSS Inc., Chicago, IL, USA). Categorical variables are expressed as counts (percentages). The distribution of the continuous variables was evaluated using the Kolmogorov-Smirnov test. Continuous variables with normal distribution are expressed as means ± standard deviations. Those with non-normal distributions are expressed as medians (interquartile ranges). Comparisons between two groups were performed using *t*-tests. The correlations between two continuous variables were assessed using Pearson's correlation analysis. For linear regression analyses, the dependent variables were the SGA, ASM/Ht^2^, HGS, and GS, and the independent variables were the PM and NPM indices. The multivariate analysis was adjusted for age, sex, and presence of DM and performed using the backward conditional method. Discrimination, which is the ability of the model to differentiate between participants who have a low GS and those who do not, was examined using the area under the receiver operating characteristic curve (AUROC). The statistical significance between the AUROCs was calculated using the DeLong method. SPSS version 25 did not compare the AUROC curves using the DeLong method. Therefore, we also used other software to compare and evaluate the statistical significance between the ROC curves. The AUROC was calculated using MedCalc version 11.6.1.0 (MedCalc, Mariakerke, Belgium). The level of statistical significance was set at *P* < 0.05.

## Results

The clinical characteristics of the 83 patients are shown in Table 1. The mean age was 56.7 ± 11.9 years; 44 (53%) of the participants were male. The dialysis duration was 3.0 (5.1) years. The mean SGA score, HGS, GS, and ASM/Ht^2^ values were 6 (2), 26.0 (9) kg, 0.92 ± 0.20 m/s, and 6.58 ± 0.98 kg/m^2^, respectively. The proportions of very low-, low-, or normal-density muscle among the entire PM area were 5.8 ± 3.1%, 17.2 ± 7.2%, and 77.0 ± 9.6%, respectively. The mean PM and NPM index values were 448.7 ± 138.7 mm^2^/m^2^ and 348.1 ± 126.3 mm^2^/m^2^, respectively.

**Table 1:** Participants' clinical characteristics.

	***N* = 83**
Male sex	44 (53%)
Age, years	56.7 ± 11.9
Diabetes mellitus	43 (51.8%)
Dialysis vintage, years	3.0 (5.1)
Hemoglobin, mg/dL	10.9 (0.7)
C-reactive protein, mg/dL	0.20 (0.40)
Blood urea nitrogen, mg/dL	59.4 ± 14.8
Creatinine, mg/dL	10.2 (2.4)
Serum calcium, mg/dL	8.4 ± 0.7
Serum phosphorus, mg/dL	5.4 ± 1.3
Serum sodium, mEq/L	138 ± 3
Serum potassium, mEq/L	5.0 ± 0.6
Serum chloride, mEq/L	98.5 ± 3.4
Intact parathyroid hormone, pg/mL	223 (240.3)
Total cholesterol, mg/dL	154 ± 34
spKt/Vurea	1.37 ± 0.31

*Data are expressed as mean ± standard deviation for continuous variables with normal distribution, median (interquartile range) for continuous variables with non-normal distribution, and as number (percentage) for categorical variables*.

Correlation coefficients for the PM index were 0.309 for the SGA score (*P* = 0.005), 0.580 for ASM/Ht^2^ (*P* < 0.001), 0.474 for HGS (*P* < 0.001), and 0.295 for GS (*P* = 0.007). Those for the NPM index were 0.338 for the SGA score (*P* = 0.002), 0.604 for ASM/Ht^2^ (*P* < 0.001), 0.537 for HGS (*P* < 0.001), and 0.384 for GS (*P* < 0.001). Because age is an important confounding factor, we also performed subgroup analyses according to 30–49, 50–59, or 60–79 years of age (Table 2). For all the groups and variables except ASM/Ht^2^ in the 30–49 age group, the correlation coefficients were greater in the NPM index than the PM index.

**Table 2:** Correlation coefficients between the PM indices and various variables according to age groups.

	**30–49 years (** ***n*** **=** **27)**				**50–59 years (** ***n*** **=** **25)**				**60–79 years (** ***n*** **=** **31)**			
	**PM index**		**NPM index**		**PM index**		**NPM index**		**PM index**		**NPM index**	
	***r***	***P***	***r***	***P***	***r***	***P***	***r***	***P***	***r***	***P***	***r***	***P***
SGA score	0.153	0.447	0.188	0.346	0.095	0.651	0.187	0.370	0.507	0.004	0.550	0.001
ASM/Ht^2^, kg/m^2^	0.637	<0.001	0.618	0.001	0.302	0.143	0.338	0.098	0.605	<0.001	0.674	<0.001
Handgrip strength, kg	0.469	0.014	0.543	0.003	0.516	0.008	0.590	0.002	0.292	0.111	0.367	0.042
Gait speed, m/s	0.455	0.017	0.500	0.008	0.087	0.680	0.189	0.366	0.096	0.607	0.260	0.158

*P-values were tested using Pearson's correlation analyses*.

*PM, psoas muscle; NPM, normal-density psoas muscle; ASM/Ht^2^, appendicular skeletal muscle mass per height squared; SGA, subjective global assessment*.

Comparisons between the low and normal groups are presented in Table 3. The PM and NPM indices in the low GS group were lower than those in the normal GS group. There were no significant differences in the PM and NPM indices between the low and normal groups for HGS, SGA, and ASM/Ht^2^, but the trends of the mean differences between the two groups were consistently greater in the NPM index than the PM index. In addition, the difference between the low and normal groups was relatively greater in HGS or GS than in ASM/Ht^2^.

**Table 3:** Comparison of the PM and NPM indices between the groups of HGS, SGA, ASM/Ht2, or GS.

	**HGS**				**SGA**			
	**Low**	**Normal**	**MD (95% CI)**	***P***	**Low**	**Normal**	**MD (95% CI)**	***P***
PM index	431 ± 146	450 ± 138	19 (−60, 99)	0.627	427 ± 117	462 ± 152	35 (−26, 96)	0.258
NPM index	308 ± 107	357 ± 129	48 (−23, 120)	0.182	321 ± 101	368 ± 140	47 (−9, 102)	0.097
	**ASM/Ht** ^**2**^				**GS**			
	**Low**	**Normal**	**MD (95% CI)**	***P***	**Low**	**Normal**	**MD (95% CI)**	***P***
PM index	423 ± 109	460 ± 152	36 (−27~100)	0.257	416 ± 124	505 ± 149	89 (28, 150)	0.005
NPM index	322 ± 92	362 ± 140	41 (−17~98)	0.161	312 ± 11	414 ± 140	102 (48, 156)	<0.001

*P-values were tested using a t-test*.

*HGS, handgrip strength; SGA, subjective global assessment; ASM/Ht^2^, appendicular skeletal muscle mass per height squared; GS, gait speed; MD, mean difference; CI, confidence interval; PM, psoas muscle; NPM, normal-density psoas muscle; PM, psoas muscle*.

**Figure 1:**
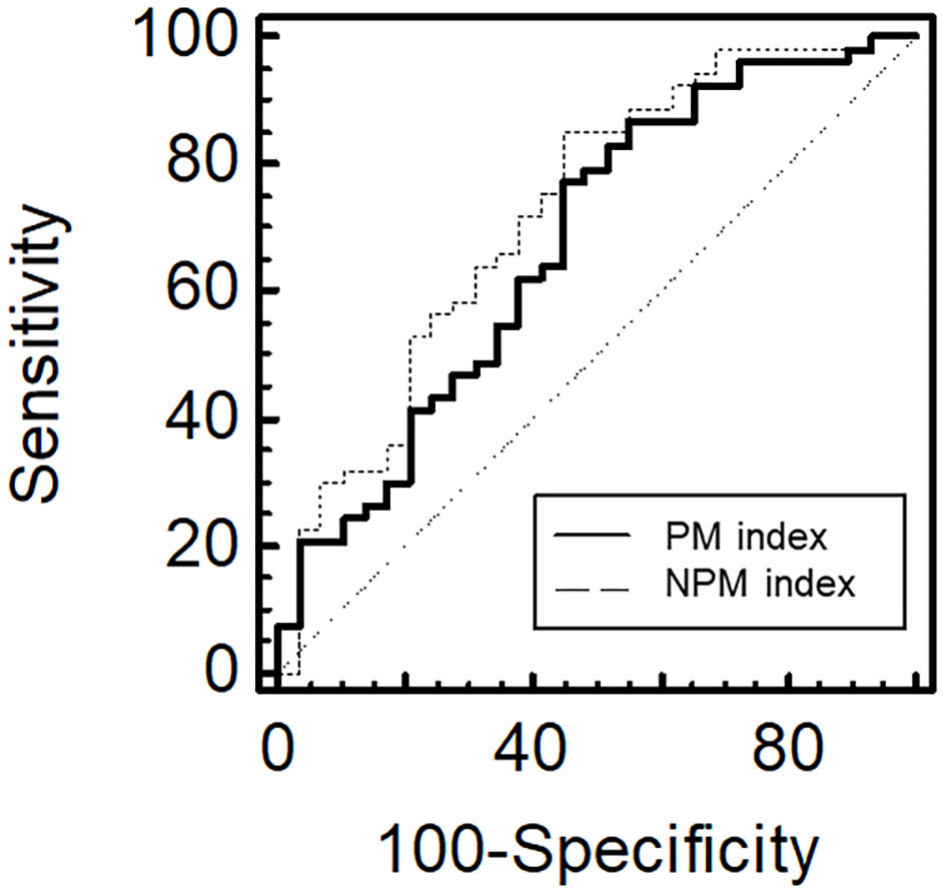
The area under the receiver operating characteristic curve for the PM or NPM indices for predicting low gait speed. NPM, normal density-psoas muscle; PM, psoas muscle.

The linear regression analysis showed that, on multivariate analysis, the NPM index was significantly associated with the SGA score, ASM/Ht^2^, and GS (Table 4). However, the PM index was significantly associated with the SGA score and ASM/Ht^2^ but not with HGS or GS. Multivariate linear regression analysis showed that statistical significance was not obtained the association between the two indices and HGS (*P* = 0.251 for PM index and *P* = 0.053 for NPM index). Non-significance between the NPM index and HGS may be associated with small sample size. However, considering lower *P*-values for NPM index compared to that for PM index, analyses with a larger sample size would lead to statistical significance between the NPM and HGS. Figure 1 shows the AUROCs for predicting the low GS of the two indices. For calculating the low GS, the AUROC values of the PM index were 0.675 [95% confidence interval (CI), 0.562–0.774; *P* = 0.007]. The AUROC values of the NPM index were 0.725 (95% CI, 0.616–0.818; *P* < 0.001). The AUROC area was significantly greater for the NPM index than for the PM index (*P* = 0.012).

**Table 4:** Comparison of nutritional and physical performance according to the PM indices.

	**PM index**				**NPM index**			
	**Univariate**		**Multivariate**		**Univariate**		**Multivariate**	
	**St-β**	***P***	**St-β**	***P***	**St-β**	***P***	**St-β**	***P***
SGA score	0.309 (0.001)	0.005	0.233 (0.001)	0.032	0.338 (0.001)	0.002	0.256 (0.001)	0.017
ASM/Ht^2^, kg/m^2^	0.580 (0.001)	<0.001	0.296 (0.001)	0.013	0.604 (0.001)	<0.001	0.380 (0.001)	0.001
Handgrip strength, kg	0.474 (0.005)	<0.001	0.136 (0.006)	0.251	0.537 (0.005)	<0.001	0.230 (0.007)	0.053
Gait speed, m/s	0.295 (0.000)	0.007	0.107 (0.000)	0.437	0.384 (0.000)	<0.001	0.298 (0.002)	0.005

*P-values were tested using linear regression analysis. The dependent variables were the SGA, ASM/Ht^2^, handgrip strength, and gait speed, and the independent variables were the PM and NPM indices. Multivariate analysis was adjusted for age, sex, and the presence of diabetes mellitus, and performed using the backward conditional method*.

*ASM/Ht^2^, appendicular skeletal muscle mass per height squared; NPM, normal-density psoas muscle; PM, psoas muscle; SGA, subjective global assessment*.

## Discussion

Previous studies have shown the importance of muscle quantity and quality in populations prone to the risk of sarcopenia and/or insulin resistance (13, 14). Muscle quality is generally associated with fat infiltration within or between the muscles. Fatty changes in the muscle can result from various conditions, such as age, obesity, DM, or surgery, that decrease muscle attenuation on CT images (15). A range of −190 HU and −30 HU is commonly used to define the fat mass on CT images. A previous study defined HU ranges of very low-, low-, or normal-density muscle of −29 to −1, 0–34, and 35–100, respectively (12). However, there were inconsistencies regarding HU attenuation cut-off values or muscle mass on CT. Low cut-off values for muscle mass ranged from −29 to 35 HU, while high cut-off values ranged from 100 to 150 HU. The wide range of HU values of muscle mass can be useful for predicting whole muscle mass area regardless of the fatty infiltration status, but a narrow range of HU values of muscle mass would be more useful for measuring the mass of muscles without fatty changes. Therefore, in our study, we used normal-density muscle mass alone to represent muscle mass without fatty infiltration.

Previous studies evaluated the association between mean PM density and prognosis in patients with various malignancies, such as melanoma, adrenocortical carcinoma, prostate cancer, and biliary or gastrointestinal tract cancer (16–20). Trotter et al. evaluated patients who underwent emergency laparotomy and showed the positive association between sarcopenia, assessed using mean PM density, and prognosis (21). Lindström et al. showed the association between PM quality and prognosis in patients treated for abdominal aortic aneurysms (22). Three studies showed a significant association between PM quality and prognosis in patients who underwent cardiac surgery (23–25). HD patients are at risk of insulin resistance. A previous study calculated the fatty changes of 49 HD patients using mean whole PM values and showed that intramuscular fat infiltration was associated with GS and 6-min walk test performance (26).

Physical performance such as GS is associated with muscle strength and/or mass (27, 28). Physical performance is also associated with various factors beyond muscle mass or strength, such as motor coordination, excitation-contraction coupling, or skeletal integrity. Therefore, decreases in physical performance can be addressed sooner than decreased muscle mass or strength. In addition, decreases in muscle strength would occur sooner than decreases in muscle mass. Although the associations among muscle mass, strength, and physical performance are complex, muscle mass with fatty changes would lead to lower strength and/or physical performance than that without fatty changes despite the same total muscle mass.

In our study, the SGA score as a nutritional index and ASM/Ht^2^ as a total skeletal muscle mass index were associated with the PM index and NPM index. ASM/Ht^2^ determined using DXA simply evaluates the total lean mass regardless of fatty changes within the muscle mass. The SGA score is a useful indicator for predicting nutritional status, but it does not discriminate whether the dominantly increased mass is muscle or fat. GS was associated with the NPM index alone. Statistical significance was not obtained, but trend showed a positive association between NPM index and HGS. Strength or physical performance can be influenced by muscle quantity and quality. Our results revealed that the NPM index as an indicator of muscle quality is more closely associated with strength or physical performance. In addition, the correlation coefficients for the SGA score, ASM/Ht^2^, HGS, and GS were greater for the NPM index than for the PM index.

Our study has inherent limitations, including a cross-sectional design with a single-center setting and a small number of patients. Our study was performed without sample size calculation according to the hypothesis. In addition, our study had a relatively too small a sample size to draw definitive conclusions from. A cross-sectional study with a relatively small sample size, such as ours, would be useful in suggesting issues regarding the associations between the variables rather than drawing definitive conclusions. Second, we did not perform multivariate analyses with adjustment for all confounding factors, such as the presence of exercise, calcium, phosphorus, i-PTH, CRP, or 25-(OH) vitamin D levels. Multivariate analyses using all confounding factors may be optimal for evaluating the independent effects of a variable. However, our study had a relatively small sample size, and we did not perform multivariate analyses adjusting for all variables. Therefore, we selected three covariates (age, sex, and the presence of DM) that can be significantly influenced by outcome measurements and performed multivariate analyses including three covariates and one variable (PM or NPM index). A prospective longitudinal study including a large number of patients and additional indicators is warranted to overcome these limitations.

In conclusion, the present study suggested that the NPM index excluding fatty infiltration may be applicable as an early and useful indicator for detecting muscle strength and physical performance among HD patients. However, limitations regarding the small sample size and the cross-sectional design without sample size calculation according to the hypothesis should be noted. Prospective studies with a larger sample size are needed to identify the definitive associations between the NPM index and muscle strength or physical performance in HD patients.

## Data Availability Statement

The raw data supporting the conclusions of this article will be made available by the authors, without undue reservation.

## Ethics Statement

The studies involving human participants were reviewed and approved by IRB of CHA Gumi medical center. The patients/participants provided their written informed consent to participate in this study.

## Author Contributions

SK conceptualized and designed the study, performed the data analysis and interpretation, and wrote the manuscript. BK and JK generated and collected the data. JD and JK drafted and revised the manuscript. All authors approved the final version of the manuscript.

## Conflict of Interest

The authors declare that the research was conducted in the absence of any commercial or financial relationships that could be construed as a potential conflict of interest.

## Publisher's Note

All claims expressed in this article are solely those of the authors and do not necessarily represent those of their affiliated organizations, or those of the publisher, the editors and the reviewers. Any product that may be evaluated in this article, or claim that may be made by its manufacturer, is not guaranteed or endorsed by the publisher.
